# Evaluation of the PP6D5 Polymer as a Novel Non-Viral Vector in the Development of a CRISPR/nCas9-Based Gene Therapy for Tay–Sachs Disease

**DOI:** 10.3390/pharmaceutics17050628

**Published:** 2025-05-09

**Authors:** Jacky M. Guerrero-Vargas, Diego A. Suarez-Garcia, Andrés F. Leal, Ivonne L. Diaz-Ariza, León D. Pérez-Pérez, Angela J. Espejo-Mojica, Carlos J. Alméciga-Díaz

**Affiliations:** 1 Institute for the Study of Inborn Errors of Metabolism, Faculty of Science, Pontificia Universidad Javeriana, Bogotá D.C. 110231, Colombia; guerrerovjackym@javeriana.edu.co (J.M.G.-V.); suarezd.i@javeriana.edu.co (D.A.S.-G.); lealb.af@javeriana.edu.co (A.F.L.); aespejo@javeriana.edu.co (A.J.E.-M.); 2 Nemours Children’s Health, Wilmington, DE 19803, USA; 3 Department of Chemistry, Universidad Nacional de Colombia, Bogotá D.C. 11001, Colombia; ildiaza@unal.edu.co (I.L.D.-A.); ldperezp@unal.edu.co (L.D.P.-P.)

**Keywords:** Tay–Sachs disease, gene therapy, CRISPR/nCas9, non-viral vectors, β-hexosaminidase A, amphiphilic graft copolymer

## Abstract

**Background/Objectives:** Tay–Sachs disease (TSD) is a neurodegenerative disorder caused by a deficiency in β-hexosaminidase A (HexA), which accumulates GM2 gangliosides, primarily in neurons. Currently, therapeutic options are limited, highlighting the need for new strategies such as gene therapy. Despite their effectiveness, viral vectors can elicit adverse immune responses; consequently, non-viral vectors are being explored as an alternative. We have previously investigated the use of CRISPR/Cas9 nickase (nCas9) as a potential tool for treating TSD. Here, we expanded our study by evaluating the PP6D5 polymer as a novel non-viral vector for delivering the CRISPR/nCas9 system to restore HexA activity. **Methods:** First, we evaluated the PP6D5-mediated CRISPR/nCas9 system’s transfection efficiency in NIH-3T3 fibroblasts, U87MG astrocytoma, SHSY5Y neuroblastoma, and TSD fibroblasts. We then evaluated the potential of PP6D5 to correct the gene defect in TSD fibroblasts. **Results:** The results showed that PP6D5 exhibited significantly higher transfection efficiency compared to lipofectamine 3000 in all tested cell models. In TSD fibroblasts, transfection with both HEXA and HEXB cDNAs increased the HexA activity levels by up to 7.4-fold, compared to a 3.2-fold increase in cells transfected only with HEXA cDNA after 15 days post-transfection. These levels were up to 4.5-fold higher than those observed in lipofectamine-mediated transfection. Additionally, PP6D5-mediated CRISPR/nCas9-based genome editing led to a significant reduction in the lysosomal mass of TSD fibroblasts. **Conclusions:** This study provides promising evidence for the use of the PP6D5 polymer as a non-viral vector for delivering CRISPR/nCas9-based gene therapy in TSD. The use of the PP6D5 polymer may offer some advantages that viral vectors cannot, such as a reduction in cytotoxicity and higher TE in difficult-to-transfect cell lines. Furthermore, this type of polymeric vector has not been extensively explored for gene therapy, making this study an important contribution to the development of non-viral delivery systems for the treatment of neurodegenerative diseases.

## 1. Introduction

Tay–Sachs disease (TSD) is a monogenic lysosomal storage disease (LSD) caused by mutations in the *HEXA* gene, which encodes the α-subunit of the lysosomal enzyme β-hexosaminidase A (HexA). HexA is a heterodimeric enzyme constituted by the α- and β-subunits, the latest encoded by the *HEXB* gene [1,2]. Mutations in the *HEXA* gene result in the loss of function of HexA, preventing the degradation of GM2 gangliosides. Lysosomal GM2 ganglioside accumulation in the central nervous system (CNS) leads to neurodegenerative symptoms in patients [1,3]. The excessive accumulation of GM2 gangliosides disrupts calcium homeostasis in the rough endoplasmic reticulum by interfering with the SERCA pump, affecting chaperones like BiP and triggering the unfolded protein response. This leads to the activation of IRE1α and PERK, which phosphorylate eIF2α, inducing ATF4 and CHOP/GADD153 expression [4], whereas CHOP promotes ERO1 activity, increasing reactive oxygen species (ROS) production. Mitochondrial proteins like BAX facilitate cytochrome C release, activating caspase 9 and the intrinsic apoptosis pathway [5,6]. In early apoptosis, phosphatidylserine externalization alters membrane asymmetry and promotes microglial recruitment [7]. Astrocyte-derived cytokines (CCL2, CXCL10) further enhance microglial activation [1,8]. These cellular changes can lead to defects in autophagy, increased lysosomal mass, ROS, synaptic dysfunction, inflammation, and ultimately cell death [1,3].

Currently, there is no specific treatment for TSD. Therefore, different therapeutic strategies are being investigated, including enzyme replacement therapy, substrate reduction therapy, and pharmacological chaperones [1,9,10]. One of the main limitations of these therapies is the blood–brain barrier (BBB), which restricts the movement of almost all molecules > 1 kDa, including recombinant proteins and gene-based drugs, preventing therapeutic molecules from crossing and performing their function [11]. Given these restrictions and the monogenic nature of this disease, different studies have focused on evaluating gene therapy (GT) alternatives for treating TSD [1]. Among the different evaluated GT strategies, genome editing approaches, such as those based on the CRISPR/Cas9 system, have emerged during the last years for the treatment of TSD [12,13].

Although the CRISPR/Cas9 system has demonstrated significant potential for biomedical applications, a critical aspect is the selection of the delivery vehicle, which prevents DNA degradation and ensures targeted delivery [14]. Various methods exist for GT delivery, yet limitations remain regarding the efficiency and safety of the vectors used. Viral vectors, such as lentivirus, adeno-associated virus, retrovirus, and adenovirus, are the most used, both in vivo and in vitro, due to their high transfection efficiency (TE) and stability. However, these vectors present challenges, including high immunogenicity, safety risks, and complex production processes [15,16]. On the other hand, non-viral vectors offer several advantages, including a high gene cargo capacity, low immunogenicity, cost-effectiveness, high safety for large-scale manufacturing, and broad cellular compatibility [17]. Common non-viral vectors used in GT include liposomes, cationic polymers, micelles, inorganic nanoparticles, and DNA nanoclews [15,18,19]. Non-viral vectors, such as polymeric or lipid nanoparticles, function by condensing and protecting DNA, facilitating its cellular delivery while preventing extracellular degradation. Electrostatic interactions between the cationic vector and DNA phosphate groups enable nanoparticle formation, enhancing cellular uptake. Polymeric nanoparticles provide greater stability and efficiency in vitro models, whereas lipid-based complexes (e.g., liposomes) enable transient transfection with diminishing gene expression over time. Understanding these differences is crucial when designing GT strategies based on the need for sustained or temporary gene expression [19,20].

Previously, we described a polymer-based non-viral vector named PP6D5 [21,22,23]. This polymer is composed of a 2 kDa segment of methoxy-poly(ethylene glycol) (mPEG), which improves the biocompatibility and colloidal stability of polymer–DNA complexes. This polymer also includes a hydrophobic backbone of poly(ε-caprolactone-*co*-propargyl carbonate) (P(CL-*co*-MPC)), which strengthens DNA interactions through hydrophobic forces. This backbone was grafted with five cationic segments of 4.4 kDa poly(2-(dimethylamino) ethyl methacrylate) (PDMAEMA) for DNA condensation [21]. PP6D5 interacts with DNA through electrostatic forces between amines, forming a complex that facilitates the delivery of GT drugs to the target cell [15]. PP6D5 formed particles with a positive surface at low N/P ratios (≥3) due to the use of several low-molecular-weight PDMAEMA grafts, which increased the charge density and DNA compaction ability. Specifically, the PP6D5 polymer complexed with DNA, at an N/P ratio of 5, showed a zeta potential of 6.6 ± 0.2 mV. It also showed a hydrodynamic diameter of 291 ± 30 nm [21,22,23]. PDMAEMA further promotes endosomal escape by protonating amines and activating the proton pump, leading to acidification of the medium, activation of enzymes and counterions, increased ionic strength, and osmotic swelling. This process ultimately disrupts the endosomal membrane, releasing DNA into the cytosol [21,22,23]. We demonstrated that the PP6D5 polymer has the capacity to transfect HEK293 and “difficult-to-transfect” Jurkat cells, achieving high expression levels of a reporter gene (i.e., EGFP) [21]. Despite its promising performance, the capacity of this polymer to mediate gene transfer within a disease context has not been evaluated, reinforcing the relevance of our study in further evaluating its potential applications.

Recently, we reported the use of a CRISPR/Cas9-based gene editing strategy that relies on a Cas9 nickase (nCas9) and the use of non-viral vectors as a potential approach for treating GM2 gangliosidoses (TS and Sandhoff diseases) [13]. The results showed the potential of CRISPR/nCas9 as a new alternative for treating GM2 gangliosidoses, as well as the capacity of non-viral vectors to mediate gene transfer for this group of disorders and for other LSDs [22,23]. In this study, we expanded our GT approach through the evaluation of the potential of the PP6D5 polymer as a non-viral vector for TSD. We evaluated the TE in several cell models, including NIH-3T3 mouse fibroblasts, U87MG astrocytoma cells, SHSY5Y neuroblastoma cells, and TSD fibroblasts, as well as the potential to correct enzyme deficiency and cellular parameters such as lysosomal mass, total reactive oxygen species, polar and neutral/apolar lipid accumulation, and autophagy. While CRISPR/nCas9-based genome editing has been demonstrated in previous studies [13,24,25], our study demonstrates the application of the PP6D5 polymer, a promising non-viral vector, for CRISPR/nCas9-based genome editing in human fibroblasts derived from TSD patients and three other different cellular models, providing a direct comparison with a gold-standard non-viral vector (Lipofectamine 3000). This comparative analysis provides valuable insights into the potential applications of PP6D5 for the development of GT for TSD.

## 2. Materials and Methods

### 2.1. PP6D5 Polymer and Plasmid DNA Preparation

**Figure 1:**
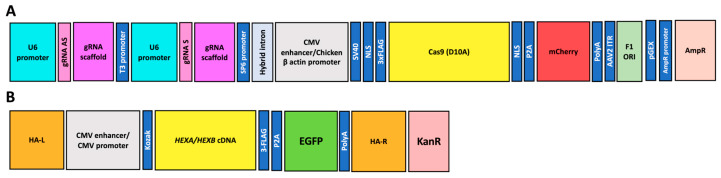
A schematic representation of the plasmid constructs used for CRISPR/nCas9-based gene editing. (**A**) A plasmid encoding nCas9, under the control of a CMV enhancer/Chicken β-actin promoter. It also contains two U6-driven guide RNAs (gRNA S and gRNA AS) for ROSA26 or AAVS1 loci, and a fluorescent marker (mCherry). Hereafter, these will be referred to as gRNA ROSA26 and gRNA AAVS1 plasmids, respectively. (**B**) A donor plasmid containing the *HEXA* or *HEXB* cDNAs, under the control of a CMV promoter, AAVS1 or ROSA26 homologous recombination arms (HA-L and HA-R), an EGFP reporter, and a KanR selection marker. Hereafter, these will be referred to as HEXA or HEXB ROSA26 and HEXA or HEXB AAVS1 plasmids, respectively.

The PP6D5 polymer was synthesized and characterized as previously described by Diaz et al. [21]. The PP6D5 polymer complexed with DNA, at an N/P ratio of 5, showed a zeta potential of 6.6 ± 0.2 mV and a hydrodynamic diameter of 291 ± 30 nm [21,22,23]. Six previously constructed plasmids [13,24,25,26] were used in this study. Two plasmids were based on the AIO-mCherry plasmid (Addgene: #74120), with one carrying Cas9 nickase (nCas9), Cas9 with a mutation in the RuvC domain (D10A), cloning sites for the sgRNA pair, an mCherry fluorescent marker, and an ampicillin resistance gene (Figure 1A). For the ROSA26 locus, gRNAs with the Sense TAAGCATGCTCTAACAGGCC and the Antisense CACAAGAGTAGTTACTTGGC were cloned onto the AIO-mCherry plasmid (hereafter, gRNA ROSA26 plasmid) [22]; meanwhile, for the AAVS1 *locus*, an sgRNA pair with the Sense ACAGACTAGAGAGGTAAAGG and the Antisense CCTGTCACGGCATCTTCCAG was cloned (hereafter, gRNA AAVS1 plasmid) [24,26]. Four plasmids containing *HEXA* or *HEXB* cDNAs, the homologous recombination arms either for ROSA26 (mouse cells, hereafter HEXA or HEXB ROSA26 plasmids) or AAVS1 (human cells, hereafter HEXA or HEXB AAVS1 plasmid) loci, an EGFP fluorescent marker, and a kanamycin resistance gene were also previously constructed (Figure 1B) [13,24,25,26]. Plasmids were prepared using the ZymoPURE II Plasmid Midiprep Kit (Zymo Research, Irvine, CA, USA), following the manufacturer’s recommendations.

### 2.2. Cell Culture

NIH-3T3 mouse embryonic fibroblasts, U87MG astrocytoma cells, a human glioblastoma cell line derived from a malignant astrocytoma in the brain of a patient, and HEK293FT were cultured in high-glucose DMEM (Biowest, Nuaillé, France) supplemented with 10% FBS and 1% antibiotics. Neuroblastoma SHSY5Y cells, a human cell line derived from a bone marrow biopsy of a neuroblastoma patient, were cultured in DMEM-F12 (Biowest) with 10% FBS and 1% antibiotics. TSD fibroblasts were obtained from the Coriell Institute, Camden, NJ, USA (GM00515); these have a c.1274_1277dupTATC mutation in the *HEXA* gene, a characteristic pathogenic insertion in exon 11 responsible for the TSD phenotype. TSD and wild-type (WT) fibroblasts, from a healthy donor, were cultured in high-glucose DMEM (Biowest, Nuaillé, France) supplemented with 15% FBS and 1% antibiotics. All cells were cultured at 37 °C with 5% CO_2_, between passages 4, 26, 30, 9, and 8 for NIH-3T3 fibroblasts, U87MG astrocytoma cells, SHSY5Y neuroblastoma cells, TSD fibroblasts, and WT fibroblasts, respectively, and were periodically tested for *Mycoplasma* spp. contamination using PCR and staining with Hoechst 33258.

### 2.3. On-Target and Homologous Recombinantion Assays

**Figure 2:**
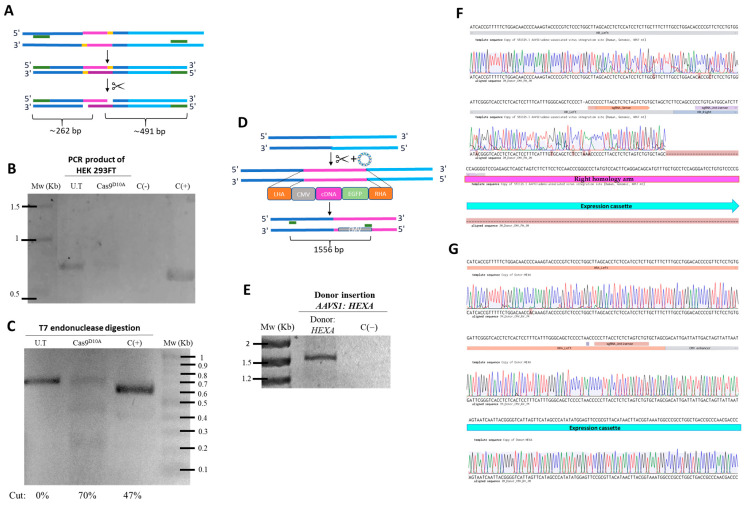
Confirmation of edition events in the genome. (**A**) The experimental design of the PCR products before and after T7 treatment. (**B**) HEK293FT cells were transfected with the gRNA AAVS1 plasmid, and a fragment flacking the on-target was amplified by PCR and visualized on 2% agarose gel. (**C**) T7 endonuclease assay results. The PCR product was digested, with heteroduplex formation of the PCR product shown when visualized on 2% agarose gel. (**D**) The experimental design showing insertion of the plasmid genomic sequence located within the homologous arms flanking a specific site at the AAVS1 locus, and amplification with specific primers to confirm integration of the sequence into the genome via homologous recombination. (**E**) HEK 293FT cells were co-transfected with both sgRNA and HEXA donor plasmids. A fragment was amplified by PCR using primers flanking part of the BHI (FW) and the CMV promoter (RV). (**F**) Sanger sequencing of the forward primer aligned with the AAVS1 locus sequence. (**G**) Sanger sequencing of the reverse primer aligned with the donor plasmid sequence AAVS1. U.T: untreated cells; C(+): positive control; C(−): negative control; Mw: molecular weight marker; HA-L: left homology arm; HA-R: right homology arm; CMV: cytomegalovirus promoter; EGFP: enhanced green fluorescent protein.

HEK 293FT cells were transfected in 24-well plates with 500 ng of each plasmid using Lipofectamine 3000 (LP; Thermo Fisher Scientific, Waltham, MA, USA), according to manufacturer’s protocol. After medium replacement, successful transfection was confirmed at 48 h via mCherry or EGFP expression using fluorescence microscopy. Genomic DNA was then extracted, with concentration, purity, and integrity assessed by NanoDrop (Thermo Fisher Scientific, Waltham, MA, USA) and 1% agarose gel electrophoresis (80 V, 1 h). The experimental design compared untransfected cells, cells with gRNA AAVS1 plasmids, or cells co-transfected with both sgRNA and HEXA donor plasmids (Figure 2).

#### 2.3.1. On-Target Assay

Genomic DNA (gDNA) was used for T7 endonuclease assays (EnGen^®^ Mutation Detection Kit, NEB, Ipswich, MA, USA), following the manufacturer’s protocol (Figure 2A). The assay was performed with gDNA from cells transfected with the gRNA AAVS1 plasmid and from untransfected cells (negative control) to detect nCas9-mediated cleavage at the AAVS1 locus. PCR amplification used the forward primer 5′-GGCCTTCTCCGACGGATGTCTCCC-3′ and the reverse primer 5′-CCGGGGGCAGGTCACGCATCCC-3′, with the following conditions: 98 °C for 30 s; 35 cycles of 98 °C for 5 s, 72 °C for 10 s, and 72 °C for 20 s/kb; and a final extension at 72 °C for 2 min. PCR products were digested with T7 endonuclease and resolved on a 2% agarose gel (80 V, 1 h) after ethidium bromide staining. Band intensities were analyzed using GelAnalyzer 19.1 to estimate cleavage efficiency by densitometry.

#### 2.3.2. Homologous Recombination Assay

To confirm HEXA cDNA insertion, PCR was performed using gDNA from cells co-transfected with both sgRNA and HEXA donor plasmids. PCR reactions used the Q5^®^ Hot Start High-Fidelity Master Mix (NEB, Ipswich, MA, USA) and primers flanking the AAVS1 locus: a forward primer targeting a region absent in the left homology arm (5′-TTCGATTGGAGTCGCTTTAACTG-3′), and reverse primer within the CMV promoter (5′-GCGGGGCCGCAGCTCTCTCTGCTTATATATAGACCTCC-3′) (Figure 2D). The PCR conditions were as follows: 98 °C for 30 s; 30 cycles of 98 °C for 10 s, 66 °C for 30 s, and 72 °C for 30 s/kb; and a final extension at 72 °C for 2 min. Amplification was confirmed on a 1% agarose gel (80 V, 1 h) with ethidium bromide staining. PCR products were Sanger sequenced (Macrogen, Seoul, Republic of Korea) and aligned to the AAVS1 locus (forward) and AAVS1 donor plasmid (reverse) using Benchling and electropherogram analysis.

### 2.4. Cell Viability Measurement with the PP6D5 Polymer

To evaluate the impact of the PP6D5 polymer on cell viability, 1 × 10^4^ cells per well were seeded in a 96-well plate. After 24 h, the PP6D5 polymer was added at concentrations ranging from 0.976 to 2000 µM and incubated for another 24 h. Then, 10 µL of MTT reagent was added to each well. After 4 h of incubation, 100 µL of dimethyl sulfoxide was added to resuspend the formazan crystals. The plate was then read in an Anthos 2020 microplate reader at 540/630 nm. IC_50_ was estimated using non-linear regression with GraphPad Prism 9.3.1 software and used to estimate IC_80_, which represents the concentration at which 80% of the maximum TE is achieved with minimal cytotoxic effects and no significant cell death, as reported previously [13,25]. All experiments were conducted in triplicate.

### 2.5. Transfection Efficiency

To evaluate the PP6D5-mediated CRISPR/nCas9 system’s TE, 5 × 10^4^ NIH-3T3 and TSD fibroblasts, and 1 × 10^5^ U87MG and SHSY5Y cells, were seeded in 24-well plates and cultured for 24 h before treatment. Untreated cells and cells transfected with empty donors served as controls. The ROSA26 sgRNA:AIO-mCherry plasmid was used for mouse cells, while the AAVS1 sgRNA:AIO-mCherry plasmid was used for human cells. Transfection was performed at a nitrogen-to-phosphate (N/P) ratio of 5:1, with 1 µg of plasmid DNA [21]. The PP6D5 polymer was prepared at 100 mM (11.7 mg in 500 µL ultrapure water), with the required amount calculated based on 5 nmol of nitrogen per 3 nmol of phosphate. Components were added sequentially (150 mM saline, DNA, polymer), vortexed for 10 s, and incubated for 20 min at room temperature [21]. The PP6D5/DNA complex was then diluted with OptiMEM™ (Gibco, Waltham, MA, USA) and added to cells after 10 min of incubation. The medium of each well was replaced with the PP6D5/DNA solution. LP transfection served as a control, following the manufacturer’s instructions. TE was assessed after 48 h by mCherry expression using an AxioScope A1 microscope (ZEISS, Goettingen, Germany). Cells were then trypsinized, centrifuged, washed with 1X PBS, and resuspended in HBSS with 1 µM propidium iodide (PI) before analysis on a BD FACSAria II cytometer (BD Biosciences, Auckland, New Zealand) using a PerCP filter (exc/emi: 670/630 nm) for mCherry and a PE filter (exc/emi: 596/578 nm) for PI detection. Data were analyzed using FlowJo vX.0.7 software. All experiments were performed in duplicate.

### 2.6. β-Hexosaminidase A Enzymatic Activity

To evaluate the effect of CRISPR/nCas9 treatment on HexA activity, 5 × 10^4^ cells/well (NIH-3T3, U87MG, SHSY5Y, and GM00515) were transfected in 24-well plates using either gRNAs and HEXA or HEXB plasmids (Figure 1). Cells treated with LP or the PP6D5 polymer without DNA, as well as with the non-viral vectors conjugated with empty donor plasmids, were included as controls. After 7, 15, and 25/30 days post transfection, cells were lysed with 150 μL of lysis buffer (0.01 M citrate-phosphate, pH 4.4, with 0.5% Triton X-100). The supernatant was collected and centrifuged for 10 min at 1600 rpm [13]. Protein quantification was performed using the BCA Protein Assay Kit (Thermo Fisher Scientific, Waltham, MA, USA). To determine the specific HexA activity, 50 µL of cell lysate supernatant was mixed with 20 µL of 3.2 μM MUGS substrate (Merck, Darmstadt, Germany) and incubated for 20 min at 37 °C. The reaction was stopped with 150 µL of stop solution (glycine-carbonate, pH 9.8). A standard curve was prepared with 4-methylumbelliferone from 0.007 to 2 µM. Fluorescence was measured at 360/445 nm (excitation/emission) using the Twinkle LB970 a fluorometer (Interchim Berthold Technologies, Montluçon, France). One unit of enzymatic activity was defined as the amount of enzyme hydrolyzing 1 nmol of substrate per hour. The specific enzymatic activity was reported as U/mg of total protein. All experiments were conducted in triplicate.

### 2.7. Measurement of Physiological Parameters by Fluorescence Microscopy

To evaluate the changes in some physiological parameters by fluorescence microscopy, coverslips were sterilized, treated with poly-D-lysine, and washed with 1X PBS. The following day, 3.3 × 10^3^ TSD fibroblasts were seeded per coverslip, incubated for 24 h, and transfected using LP or the PP6D5 polymer conjugated with nCas9 and Donor plasmids for the AAVS1 locus. Non-affected human fibroblasts and untreated TSD fibroblasts were used as controls. After 30 days, cells were stained with specific probes or transfected with reporter plasmids. To evaluate the lysosomal mass, cells were incubated with 50 nM LysoTracker™ Deep Red (Thermo Fisher Scientific, Waltham, MA, USA) in complete DMEM at 37 °C for 1 h [27]. Hoechst 33342 (Biotium, Fremont, CA, USA) (1/2000 dilution) was added and incubated for 10 min at RT. Cells were washed twice with 1X PBS and fixed with 4% paraformaldehyde, incubated at RT for 15 min, and then washed again. Cells were mounted on coverslips with Fluoroshield and observed with DAPI (Hoechst; exc/emi: 358/461 nm) and DsRed (exc/emi: 558/583 nm) filters under an AxioScope A1 microscope (ZEISS, Goettingen, Germany) with a 40X objective. To evaluate lipid accumulation, cells were incubated with 1 µM Nile Red (Thermo Fisher Scientific, Waltham, MA, USA) and Hoechst 33342 (Biotium, Fremont, CA, USA) (1/2000 dilution). Cells were treated as described for LysoTracker, and observed under an AxioScope A1 epifluorescence microscope (ZEISS, Goettingen, Germany) with a 40X objective using DAPI (nucleus), DsRed (polar lipids), and eGFP (neutral and nonpolar lipids; exc/emi: 488/509 nm) filters. Finally, to evaluate the autophagy flux, cells were transfected with the pMXs GFP-LC3-RFP plasmid (Addgene #117413). This plasmid expresses the LC3 protein fused with GFP and RFP proteins. Transfection was performed with LP 3000, and 48 h later, the wells were washed with 1X PBS, stained with Hoechst, and treated as described previously. Observations were conducted using an AxioScope A1 epifluorescence microscope with DAPI, DsRed, and eGFP filters. Autofluorescence was controlled by establishing the exposure time based on untreated cells.

### 2.8. Measurement of Physiological Parameters by Flow Cytometry

To evaluate changes in physiological parameters using flow cytometry, 5 × 10^3^ TSD fibroblasts per well were seeded in 24-well plates and transfected the following day with CRISPR/nCas9 and donor AAVS1 plasmids. After 30 days post transfection, cells were stained with probes to assess functional parameters. For lysosomal mass measurement, cells were incubated with 50 nM LysoTracker™ Deep Red (Thermo Fisher Scientific, Waltham, MA, USA) in complete DMEM at 37 °C for 1 h. For total ROS measurement, cells were incubated with 1 µM H2DCFDA (Invitrogen, Waltham, MA, USA) in complete DMEM at 37 °C for 45 min. For lipid analysis, cells were treated with 1 µM Nile Red (Invitrogen, Waltham, MA, USA) for 10 min at 37 °C. The cells were washed twice with 1X PBS, trypsinized, centrifuged, and resuspended in HBSS buffer with 1 µM PI (Thermo Fisher Scientific, Waltham, MA, USA). Fluorescence was measured using a BD FACSAria II flow cytometer (BD Biosciences, East Rutherford, NJ, USA) with PerCP (Lysotracker, Exc/Emi: 670/30 nm), FITC (ROS and neutral/apolar lipids, Exc/Em: 494/519 nm), and PE (polar lipids, Exc/Emi: 596/578 nm) filters. WT and untreated TSD fibroblasts were used as controls. Data analysis was performed using FlowJo vX.0.7 software, and all experiments were conducted in triplicate.

### 2.9. Statistical Analysis

Normality and homogeneity were assessed using the Shapiro–Wilk test. Welch’s *t*-test was used for comparisons between two treatments. One-way ANOVA with Sidak’s test was used for multiple comparisons when analyzing a single factor. For one-way ANOVA that included a control group compared with the other groups, Dunnett’s test was used instead. Two-way ANOVA with Sidak’s test was applied for comparisons involving two factors. If the data did not meet the assumptions, the Kruskal–Wallis test was used. All data were analyzed using GraphPad Prism 9.3.1 with a significance level of *p* < 0.05.

## 3. Results

In this study, we evaluated the viability, TE, and impact on HexA activity or physiological cell parameters of a CRISPR/nCas9 strategy involving transfection using the PP6D5 polymer as a novel non-viral vector in NIH-3T3 mouse embryonic fibroblasts, U87MG astrocytoma, SHSY5Y neuroblastoma, and human TSD fibroblasts (GM00515). NIH-3T3 fibroblasts were selected for evaluation of the impact of the polymer on mouse cells, as an initial approach to the future preclinical evaluation of our CRISPR/nCas9-based GT. Similarly, the U87MG astrocytoma and SHSY5Y neuroblastoma cells were included to evaluate the effect of the PP6D5-mediated CRISPR/nCas9 system’s transfection on relevant CNS-associated cells [1]. Finally, human TSD fibroblasts have been used as cellular models in drug discovery studies that have demonstrated alterations in lysosomal mass and neutral lipids [28,29].

### 3.1. Confirmation of Edition Events in the Genome

The results from cells treated with the sgRNA AAVS1 plasmid showed amplification of the targeted region (Figure 2B). After addition of T7 endonuclease, following heteroduplex formation by temperature gradient, digestion of the PCR product was observed, confirming the edition of CRISPR/nCas9 at the targeted site (Figure 2C). Digestion resulted in fragments of approximately 0.2 kb and 0.5 kb, as predicted in silico (Figure 2A), along with the parental band, with a cutoff percentage of 70% for Cas9 D10A. In contrast, amplification of gDNA from untreated cells (U.T; negative control) showed a 0.7 kb band in the AAVS1 locus region, without cutoff bands, after T7 endonuclease treatment, due to the absence of editing. The positive control produced fragments of approximately 0.2 kb and 0.4 kb after digestion, in addition to the parental band (Figure 2B).

Genomic integration of the donor cassette into the AAVS1 locus was validated using junction PCR and Sanger sequencing, confirming correct insertion via homologous recombination. Only cells that underwent homologous recombination could integrate the expression cassette into their genome, yielding a PCR product of ~1.5 kb (Figure 2D,E). Sanger sequencing confirmed the presence of the HexA expression cassette and the intended mutation in the PAM in the HA-L of amplicons from HEK293FT cells transfected with the CRISPR-nCas9 system (Figure 2F). Additionally, sequencing using the reverse primer and alignment with the HEXA AAVS1 plasmid confirmed that the insertion corresponded to the expression cassette (Figure 2G).

### 3.2. The Effect of the PP6D5 Polymer on Cell Viability and Transfection Efficiency

**Figure 3:**
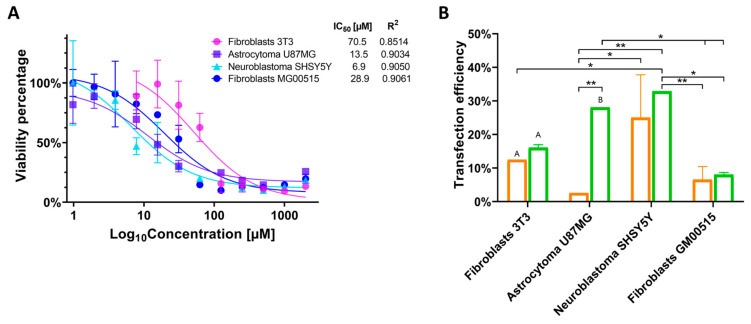
Cytotoxicity and transfection efficiency evaluation. (**A**) The effect of the PP6D5 polymer on cell viability. NIH-3T3 mouse embryonic fibroblasts, U87MG glioblastoma, SHSY5Y neuroblastoma, and human TSD fibroblasts (GM00515) were incubated with the PP6D5 polymer and the cell viability was determined through an MTT assay. IC_50_ was estimated using non-linear regression. n = 3. (**B**) Transfection efficiency was assessed using Lipofectamine 3000 (orange) and the PP6D5 polymer (green) in different cell lines using the AIO-mCherry plasmid, which contains the mCherry fluorescent reporter gene, reflecting the optimal capacity of the PP6D5 system for gene delivery. Group names: fibroblasts 3T3 (mouse fibroblasts NIH_3T3), astrocytoma U87MG, neuroblastoma SHSY5Y, fibroblasts GM00515 (TSD fibroblasts). n = 3. * *p* ≤ 0.05, ** *p* ≤ 0.01, A *p* ≤ 0.0001, B *p* ≤ 0.01, comparison with their respective controls.

Previously, the effect of the PP6D5 polymer on cell viability was evaluated in HEK293 and Jurkat cells [21]. In this study, we first determined the half-maximal inhibitory concentration (IC_50_) of the PP6D5 polymer in NIH-3T3 mouse embryonic fibroblasts, U87MG human astrocytoma cells, SHSY5Y human neuroblastoma cells, and human TSD fibroblasts. Figure 3A shows the impact of different concentrations of PP6D5 polymer on cell viability, as assessed by the MTT assay. For each cell type, non-linear regression analysis was used to calculate the PP6D5 polymer IC_50_ values, which were found to be 70.5, 13.5, 6.9, and 28.9 μM for NIH-3T3, U87MG, SHSY5Y, and TSD cells, respectively. Based on these IC_50_ values, the PP6D5 polymer IC_80_ values were calculated as 45, 7.9, 4.5, and 7 μM for NIH-3T3, U87MG, SHSY5Y, and TSD cells, respectively (Figure 3A). These IC_80_ values were then used to determine the optimal polymer quantity required for transfecting each type of cell.

Subsequently, the TE was evaluated by using the fluorescent reporter present in the AIO-mCherry plasmid (Figure 1). In NIH-3T3 fibroblasts, transfection with the PP6D5 polymer achieved a TE of 16.06%, slightly higher than that of LP (12.5%). In U87MG cells, the PP6D5 polymer demonstrated a significantly higher TE, reaching a 28.05% level, compared to 2.6% for LP-treated cells. The SHSY5Y cell line exhibited the highest TE using the PP6D5 polymer, achieving 32.85%, compared to 25.05% for LP. Finally, in TSD fibroblasts, the PP6D5 polymer outperformed LP, with TE of 8.045% and 6.565%, respectively. Overall, the PP6D5 polymer consistently showed higher TE compared to LP (Figure 3B). Moreover, there were some significant differences between the evaluated cell models.

### 3.3. The Impact of Transfection on HexA Activity

Following transfection with the PP6D5 polymer and LP, the enzyme activity was assessed at 7, 15, and 25/30 days post gene editing. For this purpose, cells were transfected with a donor plasmid containing either the HEXA cDNA or both the HEXA and HEXB cDNAs.

**Figure 4:**
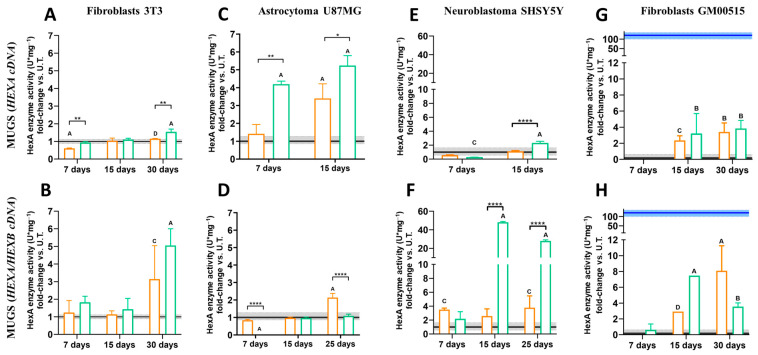
HexA enzymatic activity in transfected cells. NIH-3T3 fibroblasts, U87MG astrocytoma, SHSY5Y neuroblastoma, and TSD fibroblasts (GM00515) were transfected using Lipofectamine 3000 (orange) and the PP6D5 polymer (green) with HEXA cDNA (**A**,**C**,**E**,**G**) or HEXA/HEXB cDNAs (**B**,**D**,**F**,**H**). Enzyme activity (n = 3) is reported as the fold-change against untreated cells (U.T., n = 10). The continuous black line and gray rectangle represent the activity of the untreated cells ± SD, while the blue line and rectangle represent the activity of the wild-type fibroblasts ± SD. n = 3, * *p* ≤ 0.05, ** *p* ≤ 0.01, **** *p* ≤ 0.0001 compared to U.T. A *p* ≤ 0.0001, B *p* ≤ 0.001, C *p* ≤ 0.01, D *p* ≤ 0.05 comparison between non-viral vectors.

After 7 days post transfection, there was not an increase in the HexA activity in NIH-3T3 fibroblasts transfected with ROSA26 plasmids; instead, the activity decreased to 0.60- and 0.93-fold. This reduction was likely due to the transient effect of transfection, leading to lower enzyme production, as well as cellular stress affecting enzyme synthesis [30]. Additionally, it has been reported that overexpression under strong promoters like CMV can induce endoplasmic reticulum stress activation [26]. At 15 days post transfection, an increase in enzyme activity was observed in cells transfected with both non-viral vectors, showing a 1.03-fold increase for LP and a 1.09-fold increase for the polymer, compared to untreated cells. A notable elevation in HexA activity was observed at 30 days post transfection for PP6D5 polymer-mediated transfection, resulting in a 1.53-fold increase in HexA activity relative to untreated cells. Transfection with LP yielded a non-significant 1.15-fold increase in HexA activity in comparison to untreated cells (Figure 4A). Although transfection with both vectors led to higher HexA activity levels than that of untreated cells at 15 and 30 days post transfection, the polymer consistently allowed higher enzyme activity levels at both time points than LP. Similarly, gene editing with both HEXA/HEXB cDNAs resulted in a significant increase in HexA activity at 30 days post transfection, reaching a 3.14- and 5.05-fold increase in the cells transfected using LP and the PP6D5 polymer, respectively. Notable, higher enzyme activity levels were observed when transfecting with HEXA/HEXB cDNAs compared to those achieved with HEXA cDNA. Regardless of the transfection of HEXA or HEXA/HEXB cDNAs, the polymer-mediated transfection allowed higher enzyme activity levels at 30 days, compared to the use of LP (Figure 4B).

In the case of astrocytoma U87MG cells, gene editing with HEXA cDNA led to a significant increase in HexA activity compared to untreated cells. After 7 days, the activity was 1.41-fold and 4.19-fold higher for LP- and the polymer-treated cells, respectively, compared to untreated cells. HexA activity levels increased at 15 days post transfection, reaching a 3.39- and 5.23-fold increase for the LP- and polymer-treated cells, respectively, compared to untreated cells. Notably, at 7 and 15 days post transfection, higher HexA activity was observed in cells transfected with the PP6D5 polymer, compared to the enzyme activity levels in the cells transfected with LP (Figure 4C). However, an evaluation at 30 days post transfection was not possible, as the cells reached 100% confluence before this time point. In contrast, gene editing with HEXA/HEXB cDNAs showed a different outcome. A significant increase in HexA activity, compared to the levels observed in untreated cells, was observed only when transfection was carried out with LP, yielding a 2.1-fold increase at 25 days post transfection. The cells transfected with the polymer, on the other hand, showed a modest increase in enzyme activity (1.08-fold), barely exceeding the values of the untreated cells (Figure 4D). We did not detect enzyme activity at 7 days post-transfection with HEXA/HEXB cDNAs and the PP6D5 polymer, which could have been due to cellular stress, as many cells died after transfection [31]. In the following days, the cellular machinery may have gradually produced and accumulated enough gene expression to generate detectable activity. In addition, the cells probably adapted over time, leading to maximum gene expression and activity by day 30, as has previously been reported using the same CRISPR/nCas9 approach [13,25,26]. On the other hand, transfection with both cDNAs resulted in lower enzyme activity, which could have been due to cellular competition for transcription and translation machinery, or overloading of the cells, leading to lower expression levels compared to transfection with a single plasmid [32].

In neuroblastoma SHSY5Y cells, 7 days post transfection with the HEXA cDNA, the enzyme activity was similar to that observed in untreated cells (Figure 4E). However, 15 days post transfection, the enzyme activity increased with the use of both non-viral vectors, surpassing the levels observed in untreated cells. At this point, polymer-transfected cells reached a 2.32-fold increase in HexA activity, significantly higher than that of LP-transfected cells, which showed a 1.10-fold increase in enzyme activity (Figure 4E). Transfection with HEXA/HEXB cDNAs led to a significant increase in enzyme activity as early as day 7, surpassing the levels observed in untreated cells (Figure 4F). At this point, the increase was more pronounced in LP-transfected cells (3.50-fold increase) compared to the cells transfected with the PP6D5 polymer (2.18-fold increase). By day 15, the HexA activity in cells transfected with LP decreased to 2.56-fold, but later rose again to 3.77-fold by day 25, showing a 0.27-fold increase compared to day 7. In contrast, the enzyme activity in PP6D5 polymer-transfected cells peaked at a 48.09-fold increase on day 15, before declining to 27.92-fold by day 25, which was significantly higher than the activity levels of untreated cells (Figure 4F).

Finally, we evaluated the transfection of the CRISPR/nCas9 system in TSD fibroblasts. The HexA activity after transfection with HEXA cDNA exceeded the levels of untreated cells, reaching a peak at 15 days post transfection with both non-viral vectors (Figure 4G). However, the HexA activity reached after transfection with the PP6D5 polymer (3.20-fold) outperformed that observed in cells transfected with LP (2.34-fold) on day 15, while at 30 days, the enzyme activity levels were similar, regardless of the vector used (3.39-fold for LP and 3.82-fold for the polymer). Transfection of TSD fibroblasts with HEXA/HEXB cDNAs using the PP6D5 polymer showed that at 7 days post transfection, the HexA activity did not exceed the levels of untreated cells, while on day 15, the enzyme activity reached a 7.46-fold increase, which decreased to 3.54-fold by day 30. In contrast, the enzyme activity in LP-transfected cells showed an 8.08-fold increase, surpassing the increment observed at 30 days after transfection with the PP6D5 polymer (Figure 4H). It is important to note that, despite the fact that the transfected cells exhibited higher HexA activity levels than the untreated cells after 15 and 30 days, these levels did not reach those observed in WT fibroblasts.

### 3.4. Evaluation of Physiological Parameters in TSD Fibroblasts

**Figure 5:**
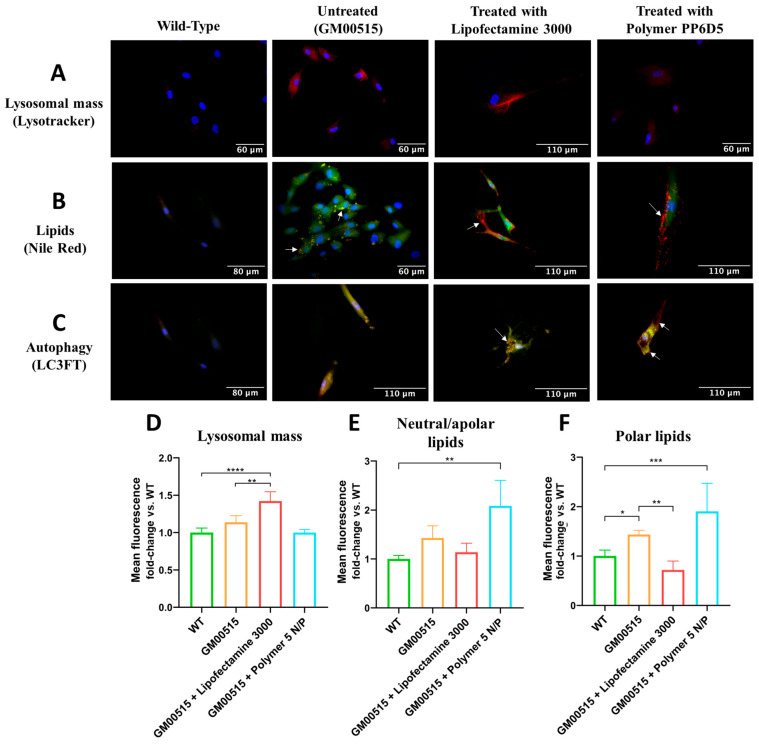
Measurement of physiological parameters in WT and TSD fibroblasts 30 days post transfection with Lipofectamine 3000 or the PP6D5 polymer. (**A**) Fluorescence images of lysosomal mass. Blue fluorescence indicates nuclear staining, and red fluorescence marks acidic compartments (lysosomes) with Lysotracker Deep Red. Images were taken with a 40X objective. (**B**) Fluorescence images of lipids. Blue fluorescence indicates nuclear staining, red fluorescence marks polar lipids, and green fluorescence marks neutral and nonpolar lipids. Images were taken with a 40X objective. (**C**) Fluorescence images of autophagy. Blue fluorescence indicates nuclear staining, red fluorescence represents the presence of the mRFP fluorescent protein, and green fluorescence represents the presence of the GFP protein, two markers used to detect autophagosomes. In the merged image, yellow fluorescence indicates the fusion of mRFP+ and GFP+, marking the presence of autophagosomes, while red fluorescence (mRFP+ only) indicates autophagic flux with the formation of autolysosomes. Images were taken with a 40X objective. (**D**) The average fluorescence of lysosomal mass, evaluated by flow cytometry. (**E**) The average fluorescence of neutral/apolar lipids, evaluated by flow cytometry. (**F**) The average fluorescence of polar lipids, evaluated by flow cytometry (n = 3). * *p* ≤ 0.05, ** *p* ≤ 0.01, *** *p* ≤ 0.001, **** *p* ≤ 0.0001.

The impact of increased enzyme activity of transfected TSD fibroblasts on lysosomal mass, total reactive oxygen species (total ROS), lipids, and autophagy was assessed. For lysosomal mass assessment, untreated TSD fibroblasts showed higher fluorescence than WT fibroblasts, suggesting lysosome accumulation. Cells treated with LP showed no significant changes, while those treated with the polymer exhibited decreased fluorescence in some fields, like WT fibroblasts (Figure 5A). Consistently, flow cytometry experiments showed that untreated TSD fibroblasts had a 1.4-fold lysosomal mass increase compared to WT fibroblasts, while PP6D5 polymer-mediated transfection led to a significant LysoTracker-associated fluorescence decrease, similar to that observed in WT fibroblasts. LP-treated TSD fibroblasts showed an increase in lysosome-associated fluorescence at 30 days post transfection (Figure 5D).

Lipid accumulation is a hallmark of GM2 gangliosidoses [28,29]. In this sense, we evaluated the levels of neutral/apolar and polar lipids in TSD fibroblasts. Figure 5B shows that untreated TSD fibroblasts exhibited increased yellow spots compared to WT fibroblasts, suggesting an increase in both polar (green) and neutral/apolar (red) lipids. Transfection with LP and the PP6D5 polymer showed clear red and green spots, representing the compartmentalization of polar and apolar lipids separately, with more pronounced red spots at the cell periphery. Additionally, flow cytometry results showed that untreated TSD fibroblasts had higher lipid accumulation compared to WT fibroblasts. LP-treated cells showed a decrease in neutral/apolar lipids (1.13 times), while polymer-treated cells showed an increase (2.08 times) compared to untreated fibroblasts (3.48 times). In LP-treated cells, polar lipids decreased (0.28 times) compared to in WT fibroblasts, while with the polymer, they showed a 0.5-fold increase (Figure 5E,F).

Finally, autophagic flux was evaluated using the LC3FT plasmid, carrying red (mRFP) and green (GFP) fluorescent proteins as markers (Figure 5C). The GFP signal is sensitive to the acidic environment of lysosomes, and is quenched upon fusion with autolysosomes, whereas the mRFP signal remains stable, allowing differentiation between autophagosomes (yellow spots: mRFP+GFP+) and autolysosomes (red spots: mRFP+). This allows for a dynamic assessment of autophagic activity by quantifying the ratio of red and yellow spots [33]. In WT fibroblasts, a dim fluorescence of both signals was observed, suggesting complete autophagic flux. In contrast, in untreated TSD fibroblasts, an increased yellow signal was observed, which suggested autophagic dysfunction. When TSD fibroblasts were transfected with the HEXA cDNA using LP, no significant change in yellow signal was observed, but red spots were identified, suggesting partial restoration of autophagic flux. In cells transfected with the polymer, the yellow intensity decreased, but yellow zones and red spots at the cell periphery were still observed, suggesting the formation of autolysosomes (Figure 5C).

Untreated TSD fibroblast cells showed no significant increase in total ROS levels compared to WT fibroblasts, limiting the analysis of the treatment with the CRISPR/nCas9 approach. Leal et al. [13] reported that, when using liposome- and magnetoliposome-assisted delivery, the most significant differences were observed in mitochondrial ROS production, rather than in global ROS levels. In this regard, a greater decrease in mitochondrial ROS was observed after LP transfection (53% reduction in TSD cells) compared to after the use of magnetoliposomes (32% reduction in TSD cells).

## 4. Discussion

We have previously explored CRISPR/nCas9-based GT approaches for LSD using non-viral vectors [13,24,25,26]. In the case of TSD, patients’ fibroblasts were transfected with HEXA cDNA by using a magnetoliposomes-based vector, and we observed not only a significant increase in β-hexosaminidase activity, but also changes in glycosaminoglycan levels, lysosome mass, and oxidative stress [13]. In this study, we expanded our GT approach through the following methods: (1) the use of the PP6D5 polymer as a non-viral vector for gene delivery in the context of TSD; (2) the evaluation of our CRISPR/nCas9-based approach in TSD relevant cells, such as astrocytoma and neuroblastoma cells; and (3) the co-transfection of HEXA and HEXB cDNAs, which was not evaluated in our previous study [13]. We investigated the use of the PP6D5 polymer, since it may offer several advantages, such as a reduction in the cytotoxicity due to the presence of PEG segments and a grafted structure with lower charge density, and a transfection improvement due to the combination of hydrophobic and cationic segments for DNA interaction [20,21]. Diaz et al. [21] demonstrated that compared to the gold-standard 25 kDa linear poly(ethylenimine), the PP6D5 polymer showed superior TE in HEK293 cells and “difficult-to-transfect” Jurkat cells. While increasing N/P ratios enhanced transgene expression, PP6D5 showed increased cytotoxicity in Jurkat suspension cells at concentrations above 20 μg/mL, with viability ranging from 35% to 80%. In these cell models, the TE never exceeded 40%, and was positively correlated with the N/P ratio and polymer concentration, but negatively correlated with the available plasmid DNA molecules, suggesting different intracellular processing mechanisms compared to adherent cells [21]. PP6D5’s superior performance over LP is likely related to its electrostatic and hydrophobic interactions, which enhance DNA binding and cellular delivery. The polymer’s flexible structure and ability to form stable DNA complexes appear to be key factors, highlighting the advantages of PP6D5 over other vectors such as LP.

Initially we demonstrated a high on-target efficiency (up to 70%) induced by CRISPR/nCas9 in cells treated with the respective plasmid, exceeding results achieved by Leal et al. [13] (36.8%) and Chiang et al. [34] (20.2%). The homologous recombination assay confirmed the successful insertion of the expression cassette into an nCas9-dependent vector, verified by PCR and Sanger sequencing, results that align with those reported previously [13,26].

Based on the IC_50_ values and TE results, incubation with the DNA–polymer complex affected the IC_50_ values differently depending on the cell type. Particularly in the U87MG astrocytoma model, the cytotoxicity increased significantly with the complete complex (polymer-CRISPR/nCas9-donor), resulting in greater cell loss than with the polymer alone. Although enzyme activity was still detected, many cells did not survive transfection, as indicated by a decrease in cell viability, whereas surviving cells retained the capacity to proliferate over time. In contrast, NIH-3T3 fibroblasts showed higher resistance, as the CRISPR/nCas9 system was less cytotoxic and did not significantly impact the initial cell population. These results suggest that both the polymer and CRISPR transfection may influence cell viability, aligning with previous studies in human pluripotent stem cells, where Cas9-induced toxicity, mediated by p53, affects cell viability [35]. Additionally, it has been reported that the cytotoxicity of cationic polymers correlates with molecular weight and charge [36]. Additionally, when forming a complex with DNA, the system’s apparent molecular weight increases, potentially affecting cytotoxicity [15,37]. Furthermore, EGFP reporter proteins in the CRISPR/nCas9-polymer complex can generate free radicals and oxidative stress, with immature eGFP producing superoxide anions and H_2_O_2_ in the presence of NADH, leading to alterations in biological pathways, including decreased HIF1α stabilization and activity [38]. Because of this, it is important to evaluate the combined effects of both the polymer and CRISPR system to optimize transfection protocols while reducing cytotoxicity.

Transfection with LP was lower across all four cellular models, and has been reported to be possibly toxic to specific cell types, leading to increased background cell death [39]. NIH-3T3 fibroblasts showed lower cytotoxicity to the PP6D5 polymer, possibly due to their stiffer extracellular matrix [36]. However, their TE was only 16.06%, lower than that of U87MG astrocytoma (28.05%) and SHSY5Y neuroblastoma (32.85%). O’Keefe et al. [37] consider a TE of ≥11.5% to be adequate, which suggests that in the present study, most cell lines were successfully transfected, except for TSD fibroblasts, which showed the lowest efficiency (8.05%), due to their primary nature limiting exogenous DNA integration and expression, though they better simulate in vivo conditions [40]. The accumulation of gangliosides in TSD cells may affect cell membrane and endocytosis mechanisms [41,42]. In contrast, Diaz et al. [21] achieved 80% transfection with PP6D5 in HEK 293FT cells, due to their more permeable membrane and faster cell cycle [43]. Since cellular characteristics influence transfection outcomes, is crucial to standardize conditions according to vector type and concentrations. For TSD, intrathecal or intracerebroventricular administration could be more effective due to the blood–brain barrier [1,44]. However, alternative strategies, like molecular Trojan horses (including engineered proteins, cell-penetrating peptides, monoclonal antibodies, and extracellular vesicles), also show potential to improve therapeutic delivery to the central nervous system [44]. The results from SHSY5Y and U87MG cell lines can help to determine appropriate polymer concentrations to minimize the risk of damage to central nervous system cells.

We evaluated HexA activity using the MUGS substrate in cells either transfected with HEXA or co-transfected with HEXA/HEXB cDNAs. When only HEXA cDNA was transfected, the heterodimer HexA (αβ) was predominantly produced, due to limited β-subunit availability, which prevented excessive formation of the more stable HexB (ββ) isoenzyme [45]. However, transfection with both HEXA and HEXB cDNAs, driven by the CMV promoter’s constitutive expression, ensured sufficient β-subunit supply, allowing balanced production of both isoenzymes [13]. At 30 days post transfection, the enzyme activity in NIH-3T3 and SHSY5Y cells was significantly increased by using the polymer and co-transfection of the HEXA and HEXB cDNAs, outperforming the results from the cells edited only with HEXA cDNA. In U87MG cells, transfection with HEXA cDNA led to the highest HexA activity levels when using both the polymer and LP, with PP6D5-transfected cells showing the highest enzyme activity during the first 7–15 days. However, by day 25, the cells transfected with HEXA/HEXB cDNAs using the polymer showed no increase in activity compared to untreated cells, suggesting possible editing inefficiency. In TSD fibroblasts, transfection with HEXA/HEXB cDNAs using LP and the PP6D5 polymer achieved 11.24% and 2.92% of the activity levels of WT fibroblasts, respectively, while the activity after transfection with HEXA cDNA alone reached 2.80% and 3.16%. In this sense, Picache et al. [46] indicate that 10–15% WT enzyme activity can reduce GM2 gangliosidosis complications. Leal et al. [13] reported an improvement in lysosomal function upon partial restoration of β-hexosaminidase A activity, reaching 10% of WT levels. This suggest therapeutic benefits from modest enzyme activity increases [47]. In SHSY5Y cells and TSD fibroblasts, transfection with HEXA/HEXB cDNAs using the PP6D5 polymer resulted in sustained enzymatic activity over time, in contrast to the previously observed decline from 15 to 30 days post transfection [13]. This persistent enzyme activity could be attributed to either stabilization of gene expression or potential genomic integration through homologous recombination. Regardless of the non-viral vector employed, the CRISPR/nCas9 system with donor DNA functioned effectively across all studied models, achieving significant increases in enzymatic activity compared to untreated cells, and demonstrating successful genomic editing.

To evaluate the impact of genomic editing on cellular alterations associated with TSD, some physiological parameters were analyzed in patient fibroblasts. Proper lysosomal function is essential for maintaining cellular homeostasis, particularly for the degradation and recycling of macromolecules. In TSD, dysfunction of the β-hexosaminidase A enzyme leads to accumulation of GM2 gangliosides, which disrupts various lysosome-related processes, including autophagy, and causes lysosomal stress [48].

The increase in lysosomal mass fluorescence in TSD fibroblasts transfected with LP may have resulted from sustained lysosomal biogenesis. This could be explained by persistent activation of TFEB, a key regulator that translocates to the nucleus under stress and upregulates lysosomal genes [47]. Wang et al. [6] observed increased nuclear TFEB in TSD models, correlating with dysfunctional lysosome accumulation, though this hypothesis requires further investigation. Previous research has demonstrated recovery of lysosomal mass in TSD fibroblasts after CRISPR/nCas9 with HEXA cDNA using LPs, but not when using MLPs, indicating that restoring lysosomal mass requires higher intracellular HexA activity production [13].

In untreated TSD fibroblasts, GM2 gangliosides and other lipids, like phospholipids and cholesterol, accumulate [1]. Nile Red (NR) staining was used to assess whether the CRISPR/nCas9 system could correct this lipid dysregulation, revealing, through fluorescence microscopy, that both polar and apolar lipids accumulate in the same cellular compartments. Previous research showed that enzyme replacement therapy with rhHex-A produced in *Komagataella phaffii* reduced lipid accumulation [28]. In the current study, lipid accumulation was evaluated after treatment with CRISPR/nCas9 using LP and the PP6D5 polymer as non-viral vectors. Cells transfected with LP showed decreased neutral/apolar lipids, likely due to partial restoration of lysosomal membrane dynamics to a level more similar to that of unaffected cells, and improved fusion with autophagosomes [48], as indicated by the lower RFP signal in Figure 5C. Meanwhile, cells transfected with the polymer showed significantly increased neutral/apolar lipids compared to WT cells. This could be related to a continuous accumulation of lipids during the period evaluated. An alternative explanation involves NR affinity for various hydrophobic structures, potentially interacting with transfection polymer residues or aggregates and overestimating lipid accumulation. This raises the possibility of interference, where NR fluorescence could be enhanced not only by lipid deposits, but also by residual polymer structures with hydrophobic characteristics [49]. These results indicate that while the polymer promotes lipid accumulation, LP facilitates the restoration of lipid balance to a level closer to that of WT cells, highlighting the importance of understanding how gene delivery strategies affect not only TE, but also broader metabolic processes, including lipid homeostasis and lysosomal dynamics.

Autophagy, which is essential for differentiation, development, and cellular homeostasis, can be compromised in neurodegenerative diseases like TSD, resulting in intracellular molecule accumulation and disrupted cellular homeostasis [50]. This study observed greater autophagosome accumulation in TSD fibroblasts, indicating disrupted lysosomal-autophagosome fusion. Settembre et al. [48] corroborate that this may result from altered lysosomal membrane lipid composition, with cholesterol and ganglioside accumulation affecting membrane dynamics and fusion capability. Similarly, increased cholesterol in late endosomes, as seen in Niemann–Pick disease, disrupts intraendosomal trafficking and membrane properties [51]. At 30 days post transfection, partial autophagy restoration was observed. Cells transfected with the PP6D5 polymer displayed higher red fluorescence signals, suggesting increased autolysosome formation and decreased lysosomal mass. This effect may be linked to the high TE achieved with the polymer, leading to enhanced therapeutic construct expression and subsequent restoration of autophagic regulation [51].

## 5. Conclusions

This study demonstrated that the PP6D5 polymer is a promising non-viral vector for GT. The PP6D5 polymer showed greater efficiency in delivering the CRISPR/nCas9 system compared to Lipofectamine 3000. Transfection with HEXA/HEXB cDNA resulted in higher enzyme activity, especially in NIH-3T3 fibroblasts, SHSY5Y neuroblastoma, and TSD fibroblasts, compared to transfection with HEXA cDNA. In these cells, the highest HexA enzyme activity was obtained after PP6D5 polymer-mediated transfection for NIH-3T3 and SHSY5Y. In TSD fibroblasts, the PP6D5 polymer reduced lysosomal mass, but did not have an impact on polar or neutral lipids, while LP had the opposite effects. To determine whether polar and neutral lipids increase due to the polymer-based therapy, it is essential to evaluate lipid storage through different analytical techniques, such as tandem mass [52]. These results suggest that CRISPR/nCas9 and the PP6D5 polymer have great potential for GT of GM2 gangliosidoses, through the co-transfection of both HEXA/HEXB cDNAs.

## Data Availability

The original contributions presented in this study are included in the article. Further inquiries can be directed to the corresponding author.

## Abbreviations

The following abbreviations are used in this manuscript:

TSD	Tay–Sachs disease
TE	Transfection efficiency
HexA	β-hexosaminidase A
HexB	β-hexosaminidase B
nCas9	Cas9 nickase
LSD	Lysosomal storage disease
CNS	Central nervous system
ROS	Reactive oxygen species
BBB	Blood–brain barrier
GT	Gene therapy
mPEG	Methoxy-poly(ethylene glycol)
P(CL-co-MPC)	Poly(ε-caprolactone-co-propargyl carbonate)
PDMAEMA	Poly(2-(dimethylamino) ethyl methacrylate)
N/P ratio	Nitrogen(polymer)/phosphate (DNA) ratio
LP	Lipofectamine 3000
IC50	Half-maximal inhibitory concentration
TFEB	Transcription factor EB
WT	Wild-type
NR	Nile Red
